# Behaviour informatics: data integration for greater understanding of human and animal behaviour

**DOI:** 10.1186/1471-2202-13-S1-P105

**Published:** 2012-07-16

**Authors:** Ansgar Koene

**Affiliations:** 1 Brain Science Institute, RIKEN, Wako-shi, Saitama 351-0198, Japan

## 

Here we propose the establishment of a new addition to the current efforts in neuro-informatics aimed at an integrated study of the manly levels of human and animal behaviour. This new platform, which we call Behaviour Informatics, will facilitate the accumulation and sharing of behavioural data, and related analysis tools. Various Neuroinformatics platforms have in recent years been established to facilitate data sharing and integration for digital atlases of brain structure and anatomy, for fMRI and electrophysiology data, for modeling of spiking neural networks and many more. These Neuroinformatics efforts promise to provide a more coherent picture of the complete brain architecture. Similar efforts in behavioural studies would facilitate a more complete understanding of the relation between behavioural traits at the micro and macro levels and their dependence on environmental conditions. In addition to the pooling and standardization of data from behavioural experiments, another pillar of behaviour informatics could be a concerted effort to use virtual environments, like massively multi player games, to gather information on human behaviour in complex dynamic (social) environments with relatively minimal effort.

In this poster I will present the goals and general framework that will form the basis of the roadmap for the creation of a Behaviour Informatics platform that will be discussed in the workshop: Behaviour Informatics: data bases, data mining and virtual world behaviour.

